# Fast Synchronization Method of Comb-Actuated MEMS Mirror Pair for LiDAR Application

**DOI:** 10.3390/mi12111292

**Published:** 2021-10-21

**Authors:** Fahu Xu, Dayong Qiao, Changfeng Xia, Xiumin Song, Yaojun He

**Affiliations:** 1 Key Laboratory of Micro/Nano Systems for Aerospace, Ministry of Education, Northwestern Polytechnical University, Xi’an 710072, China; fahu.xu@mail.nwpu.edu.cn; 2 Ningbo Institute of Northwestern Polytechnical University, Ningbo 315103, China; 3 Xi’an Zhisensor Technologies Co., Ltd., Xi’an 710000, China; xchangfeng@mail.nwpu.edu.cn (C.X.); xiumin.song@zhisensor.com (X.S.); yaojun.he@zhisensor.com (Y.H.)

**Keywords:** MEMS LiDAR, MEMS mirror pair, synchronization method

## Abstract

MEMS-based LiDAR (micro-electro–mechanical system based light detection and ranging), with a low cost and small volume, becomes a promising solution for the two-dimensional (2D) and three-dimensional (3D) optical imaging. A semi-coaxial MEMS LiDAR design, based on a synchronous MEMS mirror pair, was proposed in our early study. In this paper, we specifically reveal the synchronization method of the comb-actuated MEMS mirror pair, including the frequency, amplitude, and phase synchronization. The frequency sweeping and phase adjustment are simultaneously implemented to accelerate the MEMS mirror synchronization process. The experiment is set up and the entire synchronization process is completed within 5 s. Eventually, a one-beam MEMS LiDAR system with the synchronous MEMS mirror pair is set up and a LiDAR with a field of view (FOV) of 60°, angular resolution of 0.2°, and frame rate of 360 Hz is obtained. The experimental results verify the feasibility of the MEMS mirror synchronization method and show a promising potential application prospect for the MEMS LiDAR system.

## 1. Introduction

In recent years, light detection and ranging (LiDAR) [1,2], as a 3D optical imaging technology, has been explored extensively and received much attention in the field of autonomous driving [3,4,5], robots [6], and unmanned aerial vehicles (UAVs) [7,8]. With the development and popularization of self-driving technologies, a low-cost, small-volume, and mass-produced LiDAR is needed, especially. Nevertheless, the conventional LiDAR generally has a bulky mechanical rotary component, which leads to a large volume and high cost [9]. A micro-electro–mechanical system (MEMS) [10,11] is based LiDAR employing a small-size and fast-speed MEMS mirror, as the scanner shows a unique advantage in reducing cost and volume. Wang et al. proposed a compact 3D LiDAR, based on an electrothermal 2-axis MEMS scanner for small UAV [12]. Yoo et al. developed a uniaxial, MEMS, mirror-based LiDAR system for autonomous driving [13]. Moss and Kimoto et al. built a low-cost, compact, non-coaxial and coaxial, MEMS scanning LiDAR for robotic applications, respectively [14,15].

**Figure 1:**
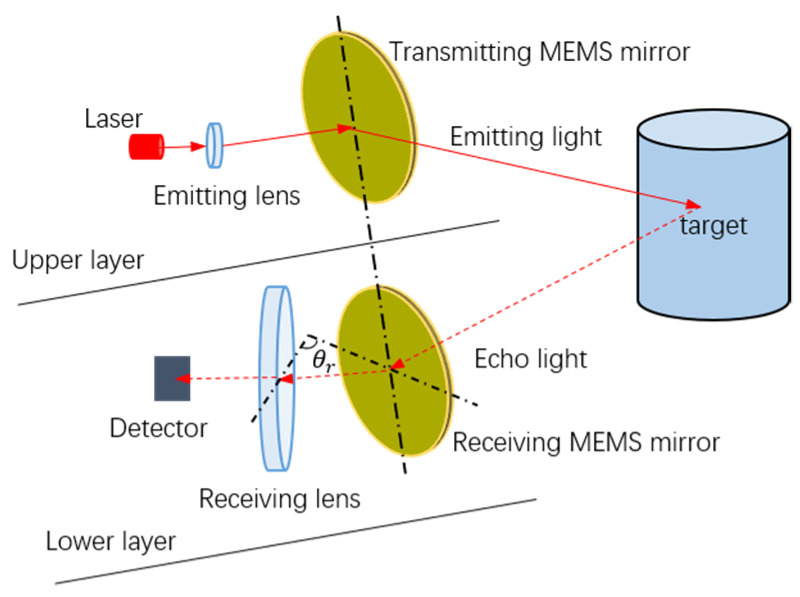
Semi-coaxial micro-electro–mechanical system (MEMS) light detection and ranging (LiDAR), based on a synchronous MEMS mirror pair.

In a MEMS-based LiDAR system, the MEMS mirror, as the crucial component, is employed to realize one-dimensional or two-dimensional (1D or 2D) scanning. However, a design trade-off exists between the scanning speed, the size, and the tilt angle of the MEMS mirror [16,17], and the current MEMS LiDAR system usually suffers from a small field of view (FOV) [18]. Much complicated optical design is typically required to achieve a large scanning FOV, which increases the complexity of the LiDAR design [19,20]. In our early study, we proposed a semi-coaxial MEMS LiDAR design, based on a synchronous MEMS mirror pair, which consists of two uniaxial MEMS mirrors, one mirror as the transmitter, and the other as the receiver [21]. This design is a two-layer structure, with the laser emitting on the upper and the echo receiving on the lower, as shown in Figure 1. Thanks to the synchronous MEMS mirror pair, the FOV of the receiving lens does not need to be taken into account, theoretically, which greatly simplifies the optical design required to achieve a large FOV. Moreover, the uniaxial MEMS mirror, with a relatively simple design and fabrication, shows a more promising potential to obtain a large FOV. For instance, Gu-Stoppel and Schwarz et al. presented a uniaxial, resonant-driven MEMS mirror, with an optical FOV of 73.2° and a mechanical scanning angle exceeding ±45°, respectively [22,23].

In order to realize a rapid start-up and response of the semi-coaxial LiDAR system, a fast synchronization of the MEMS mirror pair is needed. In this paper, two comb-actuated, uniaxial MEMS mirrors were employed, one mirror as the transmitter and the other as the receiver. We theoretically analyzed the requirements of the MEMS mirror synchronization, including the frequency synchronization, amplitude synchronization, and phase synchronization. The MEMS mirror synchronization process can be realized by using the same driving signal, compensating driving voltage, and adjusting driving phase. A fast synchronization method of the MEMS mirror pair is proposed by performing the frequency sweeping and phase adjustment at the same time, and then the amplitude adjustment. The entire synchronization procedure is also described in detail. An experiment of the MEMS mirror synchronization was carried out and the experimental result proved the feasibility of the design method for achieving a fast MEMS mirror synchronization. Eventually, a one-beam MEMS LiDAR system, based on the synchronous MEMS mirror pair, was set up, and the experiment shows a promising potential application prospect of this design.

The rest of this paper is organized as follows: Section 2 gives the theoretical analysis of the comb-actuated MEMS mirror synchronization. Section 3 detailed describes the MEMS mirror synchronization method, and the entire synchronization procedure is revealed. In Section 4, the experiments are carried out to verify the feasibility of the fast synchronization method of the MEMS mirror pair. In Section 5, the conclusion is presented.

## 2. Theoretical Analysis on MEMS Mirror Synchronization

In order to achieve the synchronization of the MEMS mirror pair, the motion characteristics of the MEMS mirror should be researched. The motion behavior of the MEMS mirror can be modeled by a second-order, nonlinear, differential equation, with a single degree of freedom (DOF), and the equation can be given by the below formulas [24,25]. Caglar and Brunner et al. also revealed the modeling formulas of the comb-actuated MEMS mirror in detail [26,27].

(1) $$I_{m}\frac{d^{2}\theta }{dt^{2}}+b\frac{d\theta }{dt}+K_{f}\theta =M\left(\theta \right)$$

(2) $$M\left(\theta \right)=-\left(r_{3}\theta^{3}+r_{1}\theta \right)V^{2}\left(\cos\left(2\pi ft+\varphi \right)+1\right)$$

In Equation (1), $I_{m}$ is the mass moment of inertia, b is the damping constant, and $K_{f}$ is the torsional stiffness of the flexures; these parameters are mainly related to the structure design and fabrication process of the MEMS mirror itself. M(θ) is the torque function, and Equation (2) shows the torque function of the electrostatic comb-drive MEMS mirror, with a squared-root sinusoid excitation signal, where $r_{3}$ and $r_{1}$ are the fitting coefficients, V is the maximum driving voltage, f is the driving frequency, and φ is the driving phase of the excitation signal. As can be seen, the dynamic vibration amplitude θ of the MEMS mirror is sufficiently related to the driving voltage, frequency, and phase of the excitation signal. Therefore, the adjustment of the excitation signal plays an important role in the realization of the MEMS mirror synchronization.

**Figure 2:**
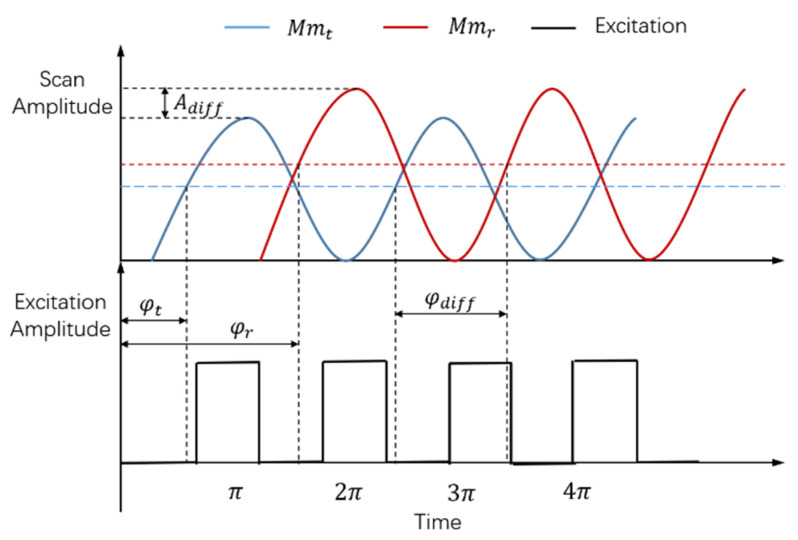
Diagram of MEMS mirror motion differences.

Actually, the motion differences of two MEMS mirrors are just manifested as the frequency difference ($f_{diff}$), amplitude difference ($A_{diff}$), and phase difference ($\varphi_{diff}$). Due to the inconsistencies of the fabrication process, differences exist between the amplitude-frequency and phase-frequency characteristics of the two MEMS mirrors, even though the structure design of the MEMS mirrors is the same. For the different amplitude-frequency characteristics, the real-time vibration amplitudes of the two MEMS mirrors are different when the same driving frequency is implemented, and vice versa. Similarly, the phase difference exists, due to the different phase-frequency characteristics of two MEMS mirrors. The phase difference firstly occurs between the excitation signal and the movement signal of the MEMS mirror itself, due to its phase-frequency characteristic. Additionally, the final phase difference of two MEMS mirrors is defined by the interval between the starting positions of two MEMS mirrors’ motion cycles. Figure 2 shows a diagram of the motion differences of two MEMS mirrors, where $Mm_{t}$ is the transmitting MEMS mirror, $Mm_{r}$ is the receiving MEMS mirror, $\varphi_{t}$ and $\varphi_{r}$ are the phase differences between the excitation signal and movement signal of the transmitting MEMS mirror and receiving MEMS mirror, and $\varphi_{diff}$ is the phase difference between two MEMS mirrors.

As demonstrated, the $A_{diff}$ and $\varphi_{diff}$ exist between two MEMS mirrors, under excitation signals with the same frequency are implemented, due to the different amplitude-frequency and phase-frequency characteristics. Therefore, the frequency synchronization, amplitude synchronization, and phase synchronization of the MEMS mirror pair are simultaneously required to achieve the final MEMS mirror synchronization. Besides, in order to realize a fast MEMS mirror synchronization, the detailed frequency, phase, and amplitude synchronization process should be researched. In addition, the MEMS mirror synchronization error should be considered in the semi-coaxial MEMS LiDAR system. As shown in Figure 1, the $\theta_{r}$ is the angle between the receiving lens and the $Mm_{r}$, so that the synchronization error affects the $\theta_{r}$ and LiDAR receiving aperture. Therefore, the LiDAR ranging distance can be affected by the synchronization error.

## 3. Synchronization Method on MEMS Mirror Pair 

For the comb-actuated MEMS mirror, a relative movement occurs between the combs, due to the electrostatic force, when a driving voltage is applied between the movable comb and fixed comb. Then, the MEMS mirror is impacted by the electrostatic force to achieve a periodic resonant rotation [28,29,30]. Based on this, driving signals with the same frequency can be implemented on the MEMS mirror pair to achieve the frequency synchronization. According to Equations (1) and (2), the difference in vibration amplitudes of the MEMS mirror pair can be compensated by adjusting the driving voltage amplitude, which contributes to the realization of the amplitude synchronization. Besides, the phase synchronization can be obtained by adjusting the phase of the driving signal. In this way, the MEMS mirror synchronization can be achieved.

Actually, more factors need to be considered, in order to achieve a fast MEMS mirror synchronization for LiDAR applications. At first, two MEMS mirrors, with similar amplitude-frequency and phase-frequency characteristics, are more conductive to achieve a fast synchronization. Typically, MEMS mirrors that are designed and fabricated on the same wafer have little differences in characteristics, which provides a potential advantage. Secondly, the selection of the MEMS mirror pair is based on the fact that the driving voltage amplitude is enough to compensate for the difference between the scanning amplitude of two MEMS mirrors when the driving signals with the same frequency are implemented. It should be noted that the high driving voltage needs the support of the hardware, and its response speed mainly determines the time of amplitude synchronization. Thirdly, a large scanning field of view (FOV) is generally required for a LiDAR application, which means that the comb-actuated MEMS mirror is preferred to work at the unstable region to obtain a larger vibration amplitude. In order to work at an unstable region, the vibration process of the MEMS mirror usually needs a frequency sweeping from a high frequency to a low frequency. Additionally, this frequency sweeping process contributes to the MEMS mirror synchronization time.

**Figure 3:**
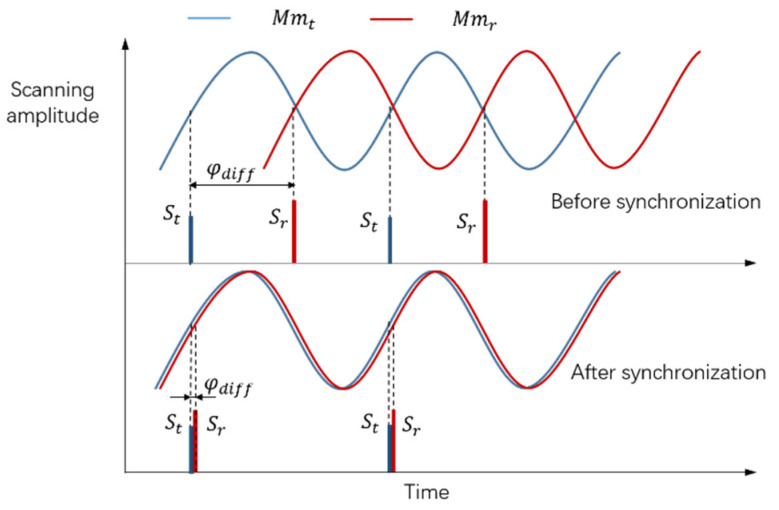
Procedure of the phase synchronization.

Moreover, the phase synchronization increases the MEMS mirror synchronization time. As mentioned above, adjusting the phase of the driving signal can gradually reduce the phase difference between two MEMS mirrors. In fact, the phase difference is detected in real time, in response to the adjustment of the driving signal. In this way, the starting signal of the MEMS mirror motion cycle is provided by the feedback control system of the MEMS mirror. Then, the phase difference can be defined by the interval between two starting signals, as shown in Figure 3. Here, $S_{t}$ is the starting signal of the transmitting MEMS mirror motion cycle, and $S_{r}$ is the starting signal of the receiving MEMS mirror motion cycle. In the phase synchronization process, gradually regulate the interval between $S_{t}$ and $S_{r}$ by adjusting the phase of driving signals, until the phase synchronization error is within the tolerance.

(3) $$T_{m}=\frac{AR}{A_{max}\times 2\pi f}$$

It should be noted that the phase synchronization error needs to be considered in this semi-coaxial design, due to its impact on the LiDAR receiving energy. The phase synchronization error should be controlled smaller than the minimum pixel interval time $T_{m}$, as defined in Equation (3). Here, AR is the LiDAR angular resolution, $A_{max}$ is the maximum mechanical amplitude of the MEMS mirror, and f is the vibration frequency of the MEMS mirror. For instance, when the frequency is 1150 Hz, the maximum mechanical amplitude is 30° and angular resolution is 0.2°; $T_{m}$ can be calculated at about 900 ns and the phase synchronization error should be lower than that. Additionally, the phase synchronization error is finally in a state of dynamic adjustment.

**Figure 4:**
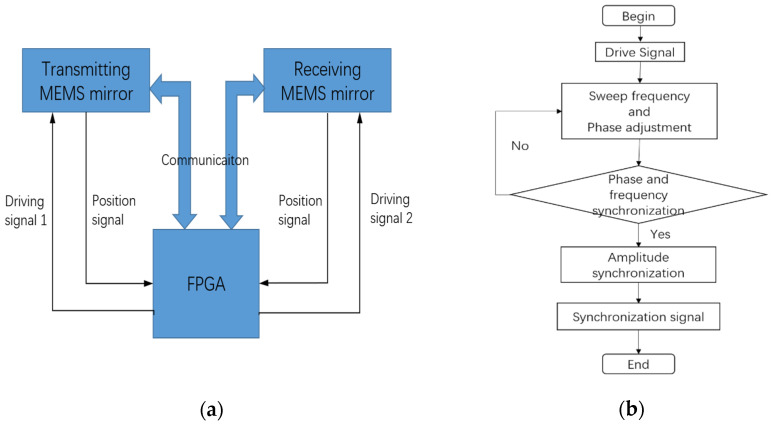
(**a**) Diagram of the MEMS mirror synchronization; (**b**) procedure of the MEMS mirror synchronization.

The frequency sweeping and phase synchronization occupy the main time of the MEMS mirror synchronization process. In order to achieve a fast MEMS mirror synchronization, the process of frequency sweeping and phase synchronization can be carried out at the same time. Figure 4a shows the diagram of the MEMS mirror synchronization. As can be seen, two drive signals with the same frequency are generated by the field programmable gate array (FPGA) and implemented to the MEMS mirror pair; the starting signals of the MEMS mirror motion cycle, generated by the MEMS mirror module, are provided to the FPGA, in order to detect the phase difference of the MEMS mirror pair. Besides, the communication between the FPGA and the MEMS mirror modules is linked by the serial peripheral interface (SPI) protocol, in order to access the state of the MEMS mirrors. The entire procedure of the MEMS mirror synchronization is depicted in Figure 4b. At first, the drive signals were implemented to force the MEMS mirrors to vibrate. Secondly, the frequency sweeping process was carried out and the phase difference was detected in real-time. It should be noted that the drive phase was real-time adjusted by FPGA to reduce the phase difference. Thirdly, the phase difference error was detected and always kept within the allowable range, until the frequency sweeping was completed. Next, the vibration amplitude of the MEMS mirror was compensated by adjusting the drive voltage to achieve the amplitude synchronization. Since then, the entire synchronization process of the MEMS mirror pair was completed, and a synchronization signal was generated to start the light detection and range.

As demonstrated above, multiple factors take time in the process of the MEMS mirror synchronization, including the process of frequency sweeping, phase synchronization, and amplitude synchronization. We analyzed these factors in detail and reveal the entire synchronization process of the MEMS mirror pair. The frequency sweeping and phase adjustment were conducted at the same time, in order to accelerate the MEMS mirror synchronization. Additionally, the synchronization error was taken into account. Finally, the synchronization of the MEMS mirror pair was completed, including the frequency synchronization, amplitude synchronization, and phase synchronization.

## 4. Experiment

**Figure 5:**
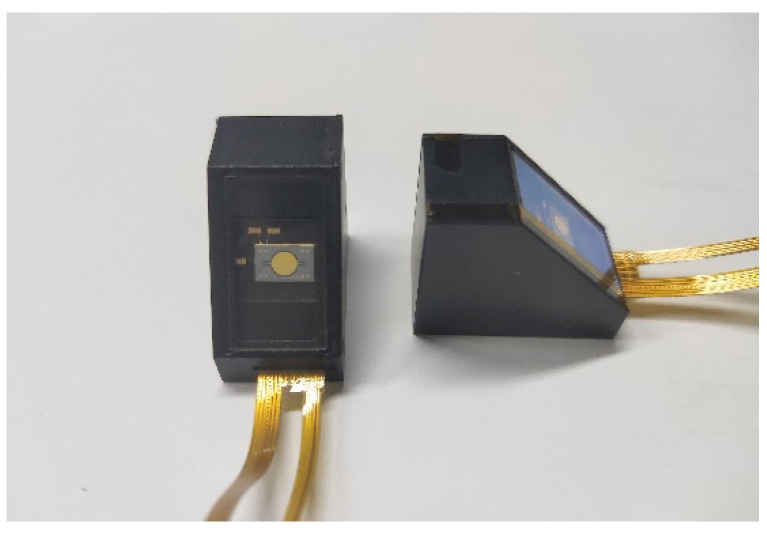
MEMS mirror modules.

In order to verify the feasibility of the synchronization method of the MEMS mirror pair for LiDAR application, the following experiment was carried out in this study. Two uniaxial MEMS mirror modules, named P1130, provided by the Zhisensor Technologies Co., Ltd. [31] (Xi’an, China), were employed as the MEMS mirror pair in this experiment. These MEMS mirror modules were integrated with the MEMS mirrors and their feedback control system. It should be noted of the that the real-time position signal MEMS mirror, with a certain angular resolution, can be provided by the MEMS mirror module [21]. The specifications of the MEMS mirror pair are presented in detail in Table 1, which shows the scanning frequency, the voltage of the drive signal, and the initial phase of the MEMS mirrors to reach the amplitude of 60°. As shown in Figure 5, the come-actuated MEMS mirrors have a diameter of 3 mm and are integrated in the MEMS mirror modules. The entire MEMS mirror module has a size of 26 mm × 15 mm × 23 mm, which contributes to the miniaturization of the MEMS LiDAR system. Besides, the maximum optical angular accuracy of the MEMS mirror is 0.05°, so that a high-angular-resolution MEMS LiDAR can be supported by these MEMS mirror modules.

**Figure 6:**
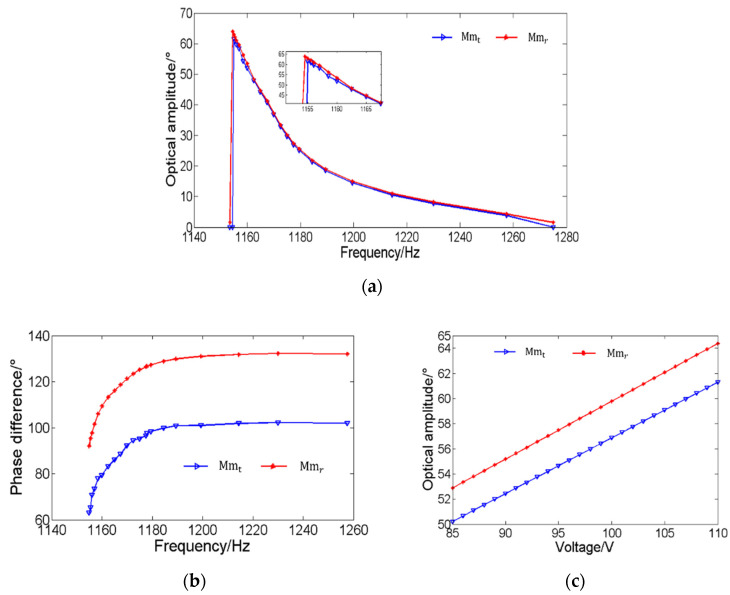
(**a**) The amplitude-frequency characteristic; (**b**) the phase-frequency characteristic; (**c**) the relationship between the scan amplitude and drive voltage.

The characteristics of the above MEMS mirror pair are investigated, in order to achieve the MEMS mirror synchronization, including the amplitude-frequency, phase-frequency, and amplitude-drive voltage characteristics. Figure 6a shows the change of the amplitude when the vibration frequency of the MEME mirror pair is from 1280 Hz to 1140 Hz. As can be seen, the optical amplitude difference between two MEMS mirrors is about 2° when the drive frequency is 1156 Hz. Figure 6b shows that the phase change trends of the two MEMS mirrors are similar, which contributes to accelerating the phase synchronization process. Figure 6c shows how the vibration amplitude changes with the drive voltage when the drive frequency is 1156 Hz, which shows the compensated drive voltage required to achieve the amplitude synchronization.

**Figure 7:**
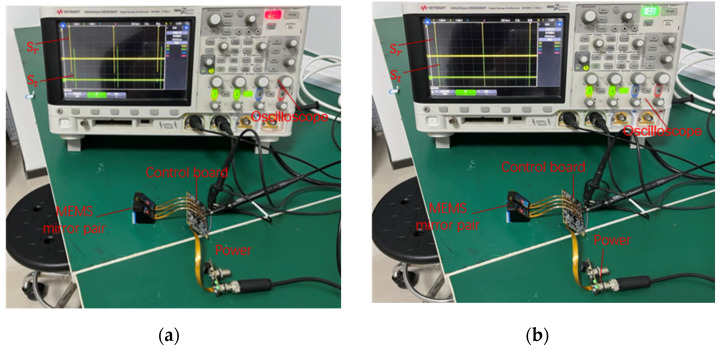
(**a**) Picture of the experiment before MEMS mirror synchronized; (**b**) picture of the experiment after MEMS mirror synchronized.

In this system, the MEMS mirror modules are connected to the main control board, which provides the power and drive signal of the MEMS mirrors. After power on, the Xilinx FPGA (Xilinx Inc., San Jose, CA, USA) outputs the drive signals to the MEMS mirror pair. A direct digital synthesizer (DDS), with a 64-bit phase accumulator, generated by the FPGA, is employed to provide the frequency sweeping signal. The whole frequency sweeping process of the MEMS mirror pair was carried out from 1280 Hz to 1156 Hz, with a speed of about 7 × 10^−6^ Hz per microsecond. Meanwhile, the MEMS mirror module provided the starting signal of the MEMS mirror motion cycle to the FPGA, in order to detect the phase difference between two MEMS mirrors. In this condition, the drive phase of the receiving MEMS mirror was adjusted by the DDS, with a speed of about 8 × 10^−6^ degrees per microsecond, to reduce the phase difference between the MEMS mirror pair. It should be noted that the phase adjustment was continuous during the whole frequency sweeping process, and the phase synchronization error was finally guaranteed within the tolerance. When the frequency sweeping process was over, the voltage of the drive signal was adjusted by the digital to analog converter (DAC), in order to reduce the amplitude difference. The amplitude synchronization process lasted less than one second. Eventually, the MEMS mirror pair works with an optical scan amplitude of 60°, a frequency of 1156 Hz, and a phase difference error within 600 ns, and the entire MEMS mirror synchronization was completed within 5 s. The entire experimental system was set up as shown in Figure 7. Additionally, the synchronization result of the MEMS mirror pair was detected on the oscilloscope.

**Figure 8:**
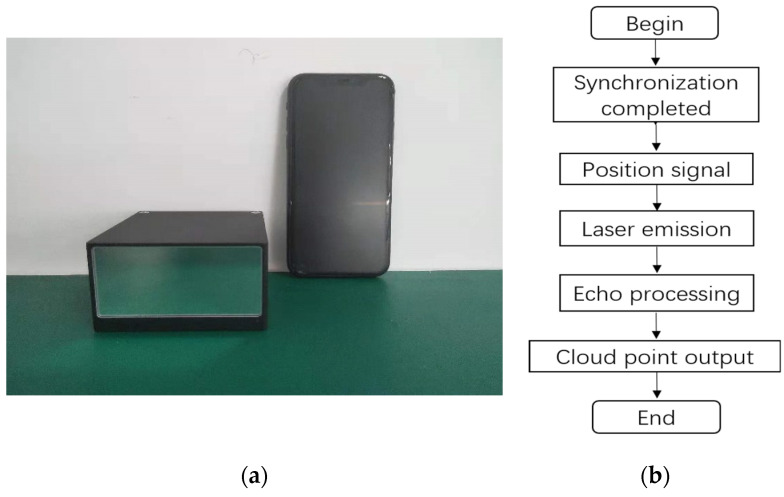
(**a**) Prototype of the MEMS LiDAR on the left and a smart phone (iPhone XR) on the right; (**b**) procedure of the MEMS LiDAR ranging process.

In order to verify the MEMS mirror synchronization design, a semi-coaxial, one-beam MEMS LiDAR system, based on the synchronous MEMS mirror pair, was set up. As shown in Figure 1, the MEMS LiDAR system consisted of two layers, the transmitting unit with the laser and transmitting MEMS mirror on the upper and the receiving unit with the avalanche photodiode (APD) detector and receiving MEMS mirror on the lower. Thanks to the miniaturizing of the MEMS mirrors and the MEMS mirror modules, the prototype of this semi-coaxial MEMS LiDAR system was built with a size of 90 mm × 90 mm × 40 mm, as shown in Figure 8a. It should be noted that the MEMS mirror modules provide the real-time position signal of the MEMS mirror, with a 0.2° angular resolution. The whole ranging process was carried out, as shown in Figure 8b. At first, the synchronization of the MEMS mirror pairs was completed. Secondly, laser pulses were emitted by the FPGA when the position signal of the MEMS mirror, with a 0.2° angular resolution, came. Then, the echo signal was converted into the electronic signal by the APD and sampled by the analog-to-digital converter (ADC). Finally, the point cloud data were generated by processing the FPGA and microcontroller unit (MCU). Benefited from the synchronous MEMS mirror pair, the MEMS LiDAR performance of a FOV of 60°, angular resolution of 0.2°, and frame rate of 360 Hz were achieved.

**Figure 9:**
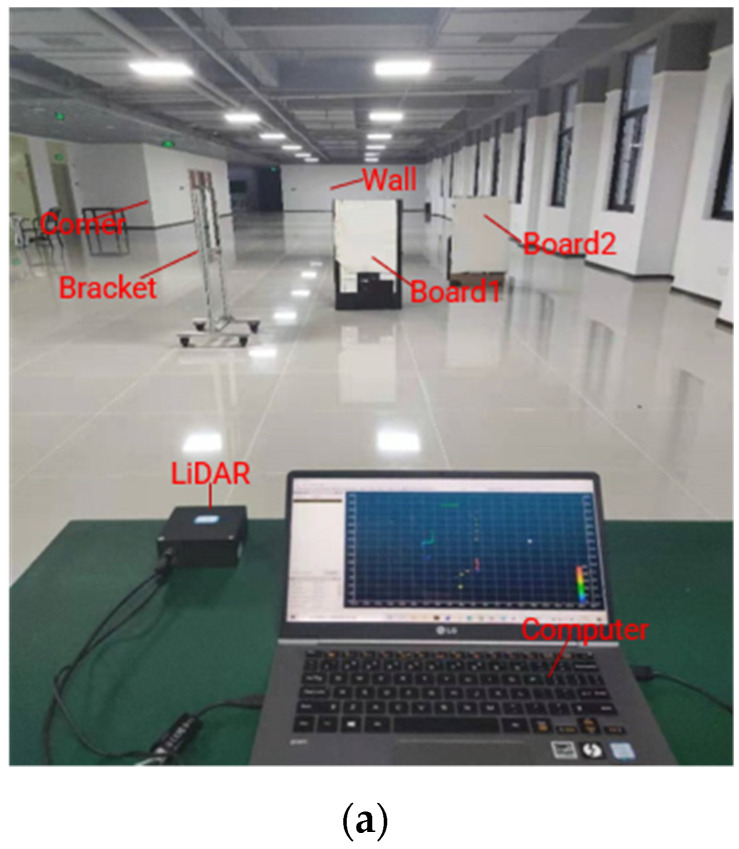

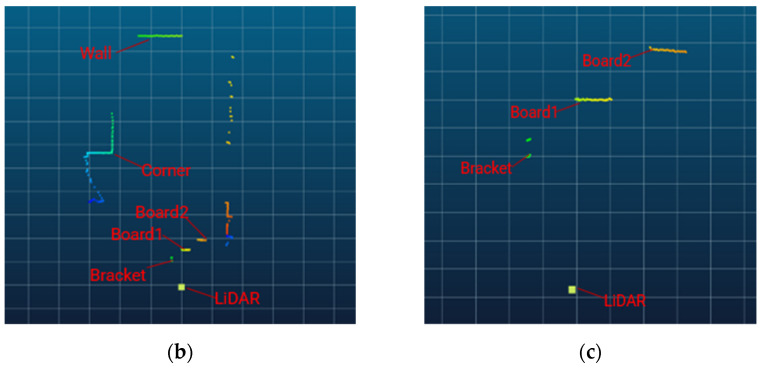
(**a**) The picture of the experiment; (**b**) the panorama of the cloud point; (**c**) the cloud point details.

Based on the above, the ranging experiment was implemented to demonstrate the imaging capability of the MEMS LiDAR system and verify the feasibility of the MEMS mirror synchronization method. When the synchronization signal of the MEMS mirror pair is given, the LiDAR system starts to work. In this experiment, a 2D point cloud image covered a 60° FOV and consisted of the wall, corner, two boards, and the bracket, as shown in Figure 9. Board 1, with a size of 0.6 m × 1.2 m, was placed approximately 5.6 m in front of this MEMS LiDAR; board 2 (0.6 m × 1.2 m) was placed at approximately 7.4 m, the bracket (with a 3 cm thickness) was placed at approximately 4.1 m, the corner was situated at about 21.1 m, and the wall was situated at approximately 37.9 m. Figure 9a shows the scene of the experiment, Figure 9b shows the panorama of the cloud point, and Figure 9c shows the cloud point details, regarding board 1, board 2, and the bracket. From the experimental results, the MEMS mirror synchronization method can be proved.

## 5. Conclusions

In this paper, we analyze the detailed requirements of MEMS mirror synchronization, including the frequency synchronization, amplitude synchronization, and phase synchronization. With the method of implementing the same drive frequency, phase adjustment, and amplitude compensation, the MEMS mirror synchronization can be achieved. The frequency sweeping and phase adjustment were simultaneously carried out, in order to accelerate the MEMS mirror synchronization process. In this way, the synchronization procedure of the MEMS mirror pair was revealed, and the experiment was set up. Two uniaxial, comb-actuated MEMS mirrors were employed in the experiment, and the entire synchronization process was completed within 5 s. A one-beam MEMS LiDAR system, with the synchronous MEMS mirror pair, was set up to verify the feasibility of the MEMS mirror synchronization method. Finally, a LiDAR, with a FOV of 60°, angular resolution of 0.2°, and frame rate of 360 Hz, was obtained, and the ranging experiment results provided the promising potential to achieve a miniaturized, large-FOV, MEMS-based LiDAR system.

## Figures and Tables

**Table 1:** Specifications of the MEMS mirror pair.

MEMS Mirror	Optical Amplitude/°	Resonant Frequency/Hz	Drive Voltage/V	Phase Difference/°	Control Accuracy/°
$Mm_{t}$	60	1156.4	100.7	102.8	0.05
$Mm_{r}$	60	1157.2	106.3	71.3	0.05
